# (*Z*)-Ethyl 2-(3-nitro­benzyl­idene)-3-oxo­butanoate

**DOI:** 10.1107/S1600536808039172

**Published:** 2008-11-26

**Authors:** Xiaopeng Shi

**Affiliations:** a Department of Chemistry and Biology, Xiangfan University, Xiangfan 441053, People’s Republic of China

## Abstract

The title mol­ecule, C_13_H_13_NO_5_, adopts a *Z* conformation at the C= C double bond. The eth­oxy atoms of the ethyl ester group are disordered over two orientations in a 3:2 ratio. Weak inter­molecular C—H⋯O hydrogen bonds help to establish the packing.

## Related literature

For applications of β-keto ester derivatives, see: Benetti *et al.* (1995 ▶); Simon *et al.* (2004 ▶). For the preparation of the title compound, see Correa & Scott (2001 ▶).
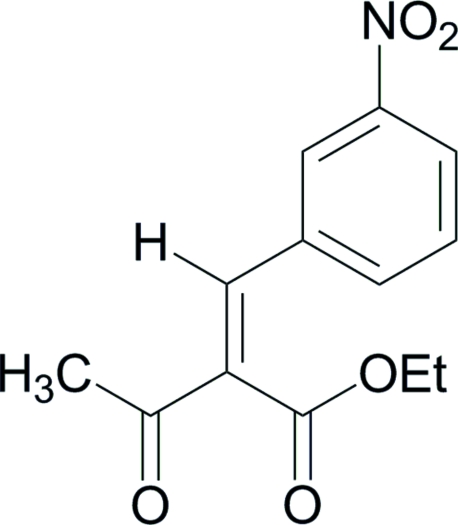


## Experimental

### 

#### Crystal data

C_13_H_13_NO_5_
*M* *_r_* = 263.24
Monoclinic, 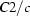

*a* = 27.6055 (6) Å
*b* = 11.8164 (2) Å
*c* = 8.2934 (1) Å
β = 102.829 (2)°
*V* = 2637.75 (8) Å^3^
*Z* = 8
Mo *K*α radiation
μ = 0.10 mm^−1^
*T* = 298 (2) K
0.20 × 0.10 × 0.10 mm

#### Data collection

Bruker SMART CCD area-detector diffractometer
Absorption correction: multi-scan (*SADABS*; Sheldrick, 1997 ▶) *T*_min_ = 0.980, *T*_max_ = 0.990
13516 measured reflections
2593 independent reflections
1793 reflections with *I* > 2σ(*I*)
*R*_int_ = 0.138

#### Refinement

*R*[*F* ^2^ > 2σ(*F* ^2^)] = 0.059
*wR*(*F* ^2^) = 0.158
*S* = 0.97
2593 reflections
194 parameters
6 restraints
H-atom parameters constrained
Δρ_max_ = 0.20 e Å^−3^
Δρ_min_ = −0.27 e Å^−3^

### 

Data collection: *SMART* (Bruker, 1997 ▶); cell refinement: *SAINT* (Bruker, 1999 ▶); data reduction: *SAINT*; program(s) used to solve structure: *SHELXS97* (Sheldrick, 2008 ▶); program(s) used to refine structure: *SHELXL97* (Sheldrick, 2008 ▶); molecular graphics: *SHELXTL* (Sheldrick, 2008 ▶); software used to prepare material for publication: *SHELXTL*.

## Supplementary Material

Crystal structure: contains datablocks I, global. DOI: 10.1107/S1600536808039172/cv2486sup1.cif

Structure factors: contains datablocks I. DOI: 10.1107/S1600536808039172/cv2486Isup2.hkl

Additional supplementary materials:  crystallographic information; 3D view; checkCIF report

## Figures and Tables

**Table 1:** Hydrogen-bond geometry (Å, °)

*D*—H⋯*A*	*D*—H	H⋯*A*	*D*⋯*A*	*D*—H⋯*A*
C4—H4⋯O2^i^	0.93	2.57	3.414 (3)	152
C6—H6⋯O3^ii^	0.93	2.49	3.381 (2)	161
C10—H10*C*⋯O4^iii^	0.96	2.44	3.350 (3)	159

Symmetry codes: (i) 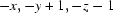; (ii) 
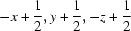
; (iii) 
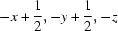
.

**Fig. 1.**
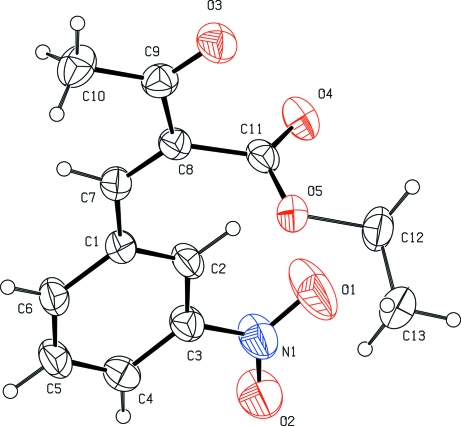
View of the title molecule showing the atom-labelling scheme. The displacement ellipsoids are drawn at the 30% probability level. Only major parts of disordered atoms are shown.

## supplementary crystallographic information

### Comment

β-Keto ester derivatives, as important synthetic intermediates, are widely applied in the synthesis of new heterocyclic derivatives presenting new pharmacological properties (Benetti *et al.*, 1995; Simon *et al.*, 2004).

The molecular structure of the title compound is shown in Fig. 1. It adopts a *Z*-conformation at the carbon-carbon double bond. The EtO atoms of the ethyl ester group are disordered over two orientations with a ratio 3:2. The molecules are connected mainly by intermolecular C—H···O interactions (Table 1).

### Experimental

The title compound was synthesized as previously described by Correa & Scott (2001) *via* Knoevenagel reaction. Colourless crystals suitable for X-ray data collection were obtained by slow evaporation of a 2:5 ratio CH_2_Cl_2_:cyclohexane solution at room temperture.

### Refinement

All H atoms were positioned geometrically (C—H = 0.93–0.97 Å) and refined as riding, allowing for free rotation of the methyl groups. The constraint *U*_iso_(H) = 1.2 *U*_eq_(C) or 1.5 *U*_eq_(C) (methyl C) was applied.

### Crystal data

**Table:**

C_13_H_13_NO_5_	*F*_000_ = 1104
*M**_r_* = 263.24	*D*_x_ = 1.326 Mg m^−^^3^
Monoclinic, *C*2/*c*	Mo *K*α radiation λ = 0.71073 Å
Hall symbol: -C 2yc	Cell parameters from 4029 reflections
*a* = 27.6055 (6) Å	θ = 2.9–22.6º
*b* = 11.8164 (2) Å	µ = 0.10 mm^−^^1^
*c* = 8.29340 (10) Å	*T* = 298 (2) K
β = 102.829 (2)º	Block, colourless
*V* = 2637.75 (8) Å^3^	0.20 × 0.10 × 0.10 mm
*Z* = 8	

### Data collection

**Table:**

Bruker SMART CCD area-detector diffractometer	2593 independent reflections
Radiation source: fine-focus sealed tube	1793 reflections with *I* > 2σ(*I*)
Monochromator: graphite	*R*_int_ = 0.138
*T* = 298(2) K	θ_max_ = 26.0º
φ and ω scans	θ_min_ = 1.5º
Absorption correction: multi-scan(SADABS; Sheldrick, 1997)	*h* = −34→30
*T*_min_ = 0.980, *T*_max_ = 0.990	*k* = −14→14
13516 measured reflections	*l* = −10→10

### Refinement

**Table:**

Refinement on *F*^2^	Secondary atom site location: difference Fourier map
Least-squares matrix: full	Hydrogen site location: inferred from neighbouring sites
*R*[*F*^2^ > 2σ(*F*^2^)] = 0.059	H-atom parameters constrained
*wR*(*F*^2^) = 0.158	*w* = 1/[σ^2^(*F*_o_^2^) + (0.0987*P*)^2^] where *P* = (*F*_o_^2^ + 2*F*_c_^2^)/3
*S* = 0.97	(Δ/σ)_max_ < 0.001
2593 reflections	Δρ_max_ = 0.20 e Å^−^^3^
194 parameters	Δρ_min_ = −0.26 e Å^−^^3^
6 restraints	Extinction correction: none
Primary atom site location: structure-invariant direct methods	

### Special details

**Table:**

Geometry. All e.s.d.'s (except the e.s.d. in the dihedral angle between two l.s. planes) are estimated using the full covariance matrix. The cell e.s.d.'s are taken into account individually in the estimation of e.s.d.'s in distances, angles and torsion angles; correlations between e.s.d.'s in cell parameters are only used when they are defined by crystal symmetry. An approximate (isotropic) treatment of cell e.s.d.'s is used for estimating e.s.d.'s involving l.s. planes.
Refinement. Refinement of *F*^2^ against ALL reflections. The weighted *R*-factor *wR* and goodness of fit *S* are based on *F*^2^, conventional *R*-factors *R* are based on *F*, with *F* set to zero for negative *F*^2^. The threshold expression of *F*^2^ > σ(*F*^2^) is used only for calculating *R*-factors(gt) *etc*. and is not relevant to the choice of reflections for refinement. *R*-factors based on *F*^2^ are statistically about twice as large as those based on *F*, and *R*- factors based on ALL data will be even larger.

### Fractional atomic coordinates and isotropic or equivalent isotropic displacement parameters (Å^2^)

**Table:**

	*x*	*y*	*z*	*U*_iso_*/*U*_eq_	Occ. (<1)
C1	0.15219 (6)	0.41046 (14)	−0.0533 (2)	0.0538 (4)	
C2	0.11986 (7)	0.33560 (15)	−0.1504 (2)	0.0614 (5)	
H2	0.1256	0.2581	−0.1407	0.074*	
C3	0.07915 (7)	0.37643 (16)	−0.2614 (2)	0.0628 (5)	
C4	0.06861 (7)	0.49032 (17)	−0.2811 (2)	0.0680 (5)	
H4	0.0404	0.5158	−0.3555	0.082*	
C5	0.10111 (8)	0.56415 (16)	−0.1875 (3)	0.0712 (6)	
H5	0.0952	0.6415	−0.1992	0.085*	
C6	0.14224 (7)	0.52611 (15)	−0.0766 (2)	0.0633 (5)	
H6	0.1641	0.5783	−0.0154	0.076*	
C7	0.19594 (6)	0.37627 (15)	0.0721 (2)	0.0560 (5)	
H7	0.2201	0.4319	0.1028	0.067*	
C8	0.20630 (6)	0.27679 (14)	0.1490 (2)	0.0527 (4)	
C9	0.25275 (7)	0.25541 (17)	0.2757 (2)	0.0606 (5)	
C10	0.28732 (8)	0.3502 (2)	0.3368 (3)	0.0772 (6)	
H10A	0.3155	0.3219	0.4159	0.116*	
H10B	0.2705	0.4060	0.3884	0.116*	
H10C	0.2982	0.3838	0.2456	0.116*	
C11	0.17337 (7)	0.17457 (15)	0.1156 (2)	0.0560 (5)	
C12	0.1062 (3)	0.0766 (7)	0.1660 (8)	0.080 (2)	0.59
H12A	0.1222	0.0066	0.2095	0.095*	0.59
H12B	0.0951	0.0698	0.0469	0.095*	0.59
C13	0.0635 (2)	0.1021 (5)	0.2428 (10)	0.116 (2)	0.59
H13A	0.0755	0.1136	0.3595	0.174*	0.59
H13B	0.0406	0.0399	0.2245	0.174*	0.59
H13C	0.0470	0.1694	0.1939	0.174*	0.59
C13'	0.0614 (2)	0.1029 (7)	0.1166 (11)	0.104 (2)	0.41
H13D	0.0468	0.1698	0.1514	0.156*	0.41
H13E	0.0393	0.0399	0.1158	0.156*	0.41
H13F	0.0668	0.1142	0.0074	0.156*	0.41
C12'	0.1100 (3)	0.0790 (8)	0.2341 (10)	0.067 (2)	0.41
H12C	0.1061	0.0758	0.3473	0.081*	0.41
H12D	0.1241	0.0082	0.2071	0.081*	0.41
N1	0.04577 (8)	0.29598 (17)	−0.3646 (2)	0.0920 (6)	
O1	0.05676 (9)	0.19660 (18)	−0.3537 (3)	0.1630 (11)	
O2	0.00831 (7)	0.33048 (16)	−0.4573 (2)	0.1147 (7)	
O3	0.26098 (5)	0.15976 (13)	0.32832 (18)	0.0838 (5)	
O4	0.17660 (6)	0.10267 (12)	0.01722 (17)	0.0800 (5)	
O5	0.14066 (5)	0.17397 (11)	0.20985 (18)	0.0728 (4)	

### Atomic displacement parameters (Å^2^)

**Table:**

	*U*^11^	*U*^22^	*U*^33^	*U*^12^	*U*^13^	*U*^23^
C1	0.0544 (10)	0.0450 (9)	0.0619 (10)	0.0003 (7)	0.0128 (8)	0.0058 (7)
C2	0.0653 (12)	0.0393 (9)	0.0735 (11)	0.0063 (8)	0.0024 (9)	0.0045 (8)
C3	0.0621 (11)	0.0530 (11)	0.0668 (10)	0.0034 (9)	0.0007 (9)	0.0049 (8)
C4	0.0647 (12)	0.0584 (12)	0.0762 (12)	0.0116 (9)	0.0056 (10)	0.0173 (9)
C5	0.0826 (14)	0.0425 (10)	0.0851 (13)	0.0066 (9)	0.0114 (11)	0.0132 (9)
C6	0.0693 (12)	0.0425 (10)	0.0757 (11)	−0.0043 (9)	0.0112 (10)	0.0082 (8)
C7	0.0508 (10)	0.0484 (10)	0.0657 (10)	−0.0028 (7)	0.0067 (8)	0.0023 (8)
C8	0.0510 (9)	0.0478 (9)	0.0574 (9)	0.0034 (7)	0.0081 (7)	0.0014 (7)
C9	0.0547 (10)	0.0651 (13)	0.0608 (10)	0.0036 (9)	0.0103 (8)	0.0054 (9)
C10	0.0618 (12)	0.0905 (16)	0.0716 (12)	−0.0141 (11)	−0.0020 (10)	0.0072 (11)
C11	0.0587 (11)	0.0434 (10)	0.0609 (10)	0.0077 (7)	0.0027 (8)	0.0047 (8)
C12	0.079 (4)	0.064 (3)	0.098 (5)	−0.026 (2)	0.023 (4)	−0.008 (3)
C13	0.076 (3)	0.080 (3)	0.200 (6)	−0.016 (3)	0.048 (4)	0.004 (5)
C13'	0.063 (4)	0.069 (4)	0.167 (7)	−0.001 (3)	−0.003 (5)	−0.006 (6)
C12'	0.075 (5)	0.052 (3)	0.075 (5)	−0.002 (3)	0.017 (4)	0.010 (4)
N1	0.0897 (14)	0.0633 (12)	0.1012 (14)	0.0036 (10)	−0.0255 (11)	−0.0002 (10)
O1	0.167 (2)	0.0637 (13)	0.196 (2)	0.0117 (12)	−0.0941 (17)	−0.0253 (12)
O2	0.0937 (12)	0.0909 (12)	0.1271 (14)	0.0037 (10)	−0.0454 (11)	0.0030 (10)
O3	0.0748 (10)	0.0686 (10)	0.0951 (10)	0.0104 (7)	−0.0090 (8)	0.0172 (8)
O4	0.1037 (12)	0.0516 (8)	0.0834 (9)	0.0019 (7)	0.0182 (8)	−0.0095 (7)
O5	0.0674 (9)	0.0496 (8)	0.1055 (10)	−0.0087 (6)	0.0277 (8)	−0.0052 (7)

### Geometric parameters (Å, °)

**Table:**

C1—C2	1.382 (2)	C10—H10C	0.9600
C1—C6	1.399 (2)	C11—O4	1.195 (2)
C1—C7	1.465 (2)	C11—O5	1.319 (2)
C2—C3	1.372 (3)	C12—O5	1.486 (6)
C2—H2	0.9300	C12—C13	1.487 (7)
C3—C4	1.379 (3)	C12—H12A	0.9700
C3—N1	1.461 (3)	C12—H12B	0.9700
C4—C5	1.364 (3)	C13—H13A	0.9600
C4—H4	0.9300	C13—H13B	0.9600
C5—C6	1.369 (3)	C13—H13C	0.9600
C5—H5	0.9300	C13'—C12'	1.500 (8)
C6—H6	0.9300	C13'—H13D	0.9600
C7—C8	1.338 (2)	C13'—H13E	0.9600
C7—H7	0.9300	C13'—H13F	0.9600
C8—C9	1.488 (3)	C12'—O5	1.446 (8)
C8—C11	1.500 (2)	C12'—H12C	0.9700
C9—O3	1.215 (2)	C12'—H12D	0.9700
C9—C10	1.485 (3)	N1—O1	1.211 (3)
C10—H10A	0.9600	N1—O2	1.213 (2)
C10—H10B	0.9600		
			
C2—C1—C6	117.59 (17)	C9—C10—H10C	109.5
C2—C1—C7	124.16 (16)	H10A—C10—H10C	109.5
C6—C1—C7	118.25 (16)	H10B—C10—H10C	109.5
C3—C2—C1	119.53 (16)	O4—C11—O5	124.43 (17)
C3—C2—H2	120.2	O4—C11—C8	124.37 (17)
C1—C2—H2	120.2	O5—C11—C8	111.19 (14)
C2—C3—C4	122.85 (18)	O5—C12—C13	105.2 (5)
C2—C3—N1	118.70 (17)	O5—C12—H12A	110.7
C4—C3—N1	118.44 (17)	C13—C12—H12A	110.7
C5—C4—C3	117.52 (17)	O5—C12—H12B	110.7
C5—C4—H4	121.2	C13—C12—H12B	110.7
C3—C4—H4	121.2	H12A—C12—H12B	108.8
C4—C5—C6	121.01 (18)	C12'—C13'—H13D	109.5
C4—C5—H5	119.5	C12'—C13'—H13E	109.5
C6—C5—H5	119.5	H13D—C13'—H13E	109.5
C5—C6—C1	121.46 (18)	C12'—C13'—H13F	109.5
C5—C6—H6	119.3	H13D—C13'—H13F	109.5
C1—C6—H6	119.3	H13E—C13'—H13F	109.5
C8—C7—C1	129.51 (17)	O5—C12'—C13'	103.4 (6)
C8—C7—H7	115.2	O5—C12'—H12C	111.1
C1—C7—H7	115.2	C13'—C12'—H12C	111.1
C7—C8—C9	123.05 (17)	O5—C12'—H12D	111.1
C7—C8—C11	124.25 (16)	C13'—C12'—H12D	111.1
C9—C8—C11	112.68 (15)	H12C—C12'—H12D	109.1
O3—C9—C10	121.61 (18)	O1—N1—O2	122.5 (2)
O3—C9—C8	118.35 (17)	O1—N1—C3	118.14 (19)
C10—C9—C8	120.03 (17)	O2—N1—C3	119.4 (2)
C9—C10—H10A	109.5	C11—O5—C12'	125.8 (4)
C9—C10—H10B	109.5	C11—O5—C12	110.2 (3)
H10A—C10—H10B	109.5		
			
C6—C1—C2—C3	−1.6 (3)	C11—C8—C9—C10	−173.76 (16)
C7—C1—C2—C3	178.23 (17)	C7—C8—C11—O4	91.9 (2)
C1—C2—C3—C4	−0.1 (3)	C9—C8—C11—O4	−87.0 (2)
C1—C2—C3—N1	179.14 (17)	C7—C8—C11—O5	−88.6 (2)
C2—C3—C4—C5	1.4 (3)	C9—C8—C11—O5	92.48 (17)
N1—C3—C4—C5	−177.85 (18)	C2—C3—N1—O1	−3.5 (3)
C3—C4—C5—C6	−0.9 (3)	C4—C3—N1—O1	175.8 (2)
C4—C5—C6—C1	−0.9 (3)	C2—C3—N1—O2	176.3 (2)
C2—C1—C6—C5	2.1 (3)	C4—C3—N1—O2	−4.4 (3)
C7—C1—C6—C5	−177.72 (17)	O4—C11—O5—C12'	12.4 (5)
C2—C1—C7—C8	−20.0 (3)	C8—C11—O5—C12'	−167.1 (4)
C6—C1—C7—C8	159.84 (18)	O4—C11—O5—C12	−4.6 (4)
C1—C7—C8—C9	−179.60 (16)	C8—C11—O5—C12	175.9 (4)
C1—C7—C8—C11	1.6 (3)	C13'—C12'—O5—C11	−98.1 (7)
C7—C8—C9—O3	−173.65 (17)	C13'—C12'—O5—C12	−49.8 (16)
C11—C8—C9—O3	5.3 (2)	C13—C12—O5—C11	−164.4 (4)
C7—C8—C9—C10	7.3 (3)	C13—C12—O5—C12'	55.7 (17)

### Hydrogen-bond geometry (Å, °)

**Table:**

*D*—H···*A*	*D*—H	H···*A*	*D*···*A*	*D*—H···*A*
C4—H4···O2^i^	0.93	2.57	3.414 (3)	152
C6—H6···O3^ii^	0.93	2.49	3.381 (2)	161
C10—H10C···O4^iii^	0.96	2.44	3.350 (3)	159

Symmetry codes: (i) −*x*, −*y*+1, −*z*−1; (ii) −*x*+1/2, *y*+1/2, −*z*+1/2; (iii) −*x*+1/2, −*y*+1/2, −*z*.
